# Antimicrobial peptide-loaded gold nanoparticle-DNA aptamer conjugates as highly effective antibacterial therapeutics against *Vibrio vulnificus*

**DOI:** 10.1038/s41598-017-14127-z

**Published:** 2017-10-19

**Authors:** Boeun Lee, Jonggwan Park, Minkyung Ryu, Soochan Kim, Minju Joo, Ji-Hyun Yeom, Suk Kim, Yoonkyung Park, Kangseok Lee, Jeehyeon Bae

**Affiliations:** 1 Department of Life Science, Chung-Ang University, Seoul, 06974 South Korea; 2 Department of Bioinformatics, Kongju National University, Kongju, 38065 South Korea; 3 Department of Biomedical Science, Chosun University, Gwangju, 61452 South Korea; 4 Institute of Animal Medicine, College of Veterinary Medicine, Gyeongsang National University, Jinju, 52828 South Korea; 5 School of Pharmacy, Chung-Ang University, Seoul, 06974 South Korea

## Abstract

*Vibrio vulnificus* causes fatal infections in humans, and antibiotics are commonly used in treatment regimens against *V. vulnificus* infection. However, the therapeutic effects of antibiotics are limited by multidrug resistance. In this study, we demonstrated that an antimicrobial peptide (AMP), HPA3P^His^, loaded onto a gold nanoparticle-DNA aptamer (AuNP-Apt) conjugate (AuNP-Apt-HPA3P^His^) is an effective therapeutic tool against *V. vulnificus* infection *in vivo* in mice. HPA3P^His^ induced bacterial cell death through the disruption of membrane integrity of *V. vulnificus*. The introduction of AuNP-Apt-HPA3P^His^ into *V. vulnificus*-infected HeLa cells dramatically reduced intracellular *V. vulnificus* by 90%, leading to an increase in the viability of the infected cells. Moreover, when *V. vulnificus*-infected mice were intravenously injected with AuNP-Apt-HPA3P^His^, a complete inhibition of *V. vulnificus* colonization was observed in the mouse organs, leading to a 100% survival rate among the treated mice, whereas all the control mice died within 40 hours of being infected. Therefore, this study demonstrated the potential of an AMP delivered by AuNP-Apt as an effective and rapid treatment option against infection caused by a major pathogen in humans and aquatic animals.

## Introduction

The emergence and spread of multidrug-resistant bacteria are global concerns. Overcoming antibiotic resistance is an urgent and imperative issue. A major hurdle in the elimination of pathogens in a host is the poor uptake of many antibiotics by infected host cells, which results in intracellular persistence of bacteria and failure of antimicrobial therapy^1–3^.

*Vibrio vulnificus*, a halophilic Gram-negative bacillus, is a highly virulent, opportunistic pathogen that causes gastroenteritis, primary sepsis, and wound infection in humans. Infection by *V. vulnificus* commonly occurs via ingestion of contaminated seafood and an exposed open wound, and its incidence is significantly rising^4^. *V. vulnificus* spreads rapidly and causes extensive tissue damage, leading to a mortality rate of over 50% among infected patients with sepsis^5^. *V. vulnificus* infects a host via gastrointestinal tract^6^, and disrupts tight junctions of intestinal cells, leading to its intracellular colonization in the intestine intestinal epithelium^7^. Combinational antibiotics therapy is the most common treatment regimen against *V. vulnificus* infection^8,9^. However, resistance to antibiotics and inefficient delivery of antibiotics contribute to the high fatality rate observed following *V. vulnificus* infection^2,4,9,10^. Therefore, innovative approaches for developing alternative antibacterial agents are in demand.

Diverse classes of antibacterial compounds have been designed for effective treatment of fatal bacterial infections. Among them, antimicrobial peptides (AMPs), which are a crucial part of the host defense system with broad-spectrum antimicrobial activity, are prospective drug candidates^11,12^. Complex mechanisms of action of AMPs decrease the potential for emergence of AMP-resistant pathogens^13^. HP(2-20), a 19-amino-acid peptide (AKKVFKRLEKLFSKIQNDK) derived from the N-terminus of *Helicobacter pylori* ribosomal protein L1 (RpL1), shows broad-spectrum antimicrobial activity against bacteria, fungi, and protozoa without causing hemolysis^14,15^. HPA3P, a derivative of HP(2-20) with substitutions of E9P, Q16W, and D18W, exhibits antimicrobial activity against *Escherichia coli*, *Staphylococcus aureus*, *Pseudomonas Aeruginosa*, *Bacillus subtilis*, and *Salmonella typhimurium* via membrane penetration^16^.

The instability of peptides *in vivo* and low penetrability in host cells are the major limiting factors for the clinical application of AMPs^17^. To overcome these limitations, we developed an AMP delivery system using gold nanoparticle-DNA aptamer (AuNP-Apt) conjugate. AuNPs have low-toxicity and non-immnogenicity^18,19^, and our previous studies indicate that the AuNP-Apt conjugates efficiently deliver proteins and peptides, maintaining their intact structure and function, into diverse types of cells^20–22^. In addition, the elimination of intracellular *Salmonella enterica* serovar Typhimurium in host cells by AuNP-Apt-conjugated AMP has been demonstrated^21^. In the present study, we investigated the effectiveness of a *Helicobacter pylori*-derived AMP, HPA3P, delivered by AuNP-Apt against *V. vulnificus* infection, which involved mechanisms of infection and virulence different from those of *S. typhimurium*. We found that the *in vivo* delivery of HPA3P loaded on AuNP-Apt effectively inhibited *V. vulnificus*, leading to 100% survival rate among *V. vulnificus*-infected mice.

## Results

### Preparation of hexahistidine-tagged HPA3P (HPA3P^His^) and antimicrobial mechanism of HPA3P^His^ against *V. vulnificus*

Three different AMPs, HPA3P, HPA3P2, and A3-APO, were tested for their bactericidal activities against *V. vulnificus*, *S. typhimurium*, or *S. aureus* by measuring their minimal inhibitory concentrations (MICs) and minimal bactericidal activity (MBC). Among them, HPA3P exhibited the lowest MIC and MBC for *V. vulnificus* and showed similar or variable MICs and MBCs for other gram-negative or -positive bacteria (Table 1). For The C-terminal end of HPA3P was tagged with hexahistidine (His) to load HPA3P on the AuNP-Apt^His^ conjugate by interaction of His aptamer (Apt^His^) with His-tagged HPA3P (HPA3P^His^). This His-tagging to HPA3P did not alter the MIC and MBC against *V. vulnificus* (Table 1), and AuNP-Apt^His^ itself exhibited no bactericidal activity up to 10 nM. The antibacterial activity of these AMPs was attributable to their bactericidal activity rather than their bacteriostatic property as indicated by similar MIC and MBC levels for three bacterial species tested in this study (Table 1). When compared with the MIC and MBC of antibiotics for *V. vulnificus*, the antibacterial activity of HPA3P^His^ was more potent than ampicillin and weaker than doxycycline and erythromycin (Table 1).

**Table 1:** Antibacterial activities of synthetic peptides and and antibiotics.

Peptides	Sequences	M. W^a^	MIC^b^/MBC^c^ (μM)		
			Vv	St	Sa
HPA3P	AKKVFKRLPKLFSKIWNWK-NH_2_	2417.2	8/8	8/8	16/16
HPA3P2	AKKVFKRLPKLPSKIWNWK-NH_2_	2367.0	>64/>64	>64/>64	>64/>64
A3-APO	RPDKPRPYLPRPRPPRPVR-NH_2_	2350	>64/>64	32/32	>64/>64
HPA3P^His^	AKKVFKRLPKLFSKIWNWKHHHHHH-NH_2_	3348.1	8/8	32/32	16/16
HPA3P2^His^	AKKVFKRLPKLPSKIWNWKHHHHHH-NH_2_	3280.9	64/64	>64/>64	>64/>64
A3-APO^His^	RPDKPRPYLPRPRPPRPVRHHHHHH-NH_2_	3480.7	>64/>64	32/32	>64/>64
**Antibiotics**	**Classification**	**M. W** ^**a**^	**MIC** ^**b**^ **/MBC** ^**c**^ **(μM)**		
			**Vv**	**St**	**Sa**
Ampicillin	Penicillins	349.4	>64/>64	8/8	1/1
Doxycycline	Tetracyclins	444.4	0.5/0.5	8/16	1/2
Erythromycin	Macrolides	733.9	2/4	>64/>64	0.5/1

^a^M.W: molecular weight.

^b^MIC: minimal inhibitory concentration against *Vibrio vulnificus* (Vv), *Salmonella typhimurium* (St), and *Staphylococcus aureus* (Sa).

^c^MBC: minimal bactericidal concentration against Vv, St, and Sa.

**Figure 1:**
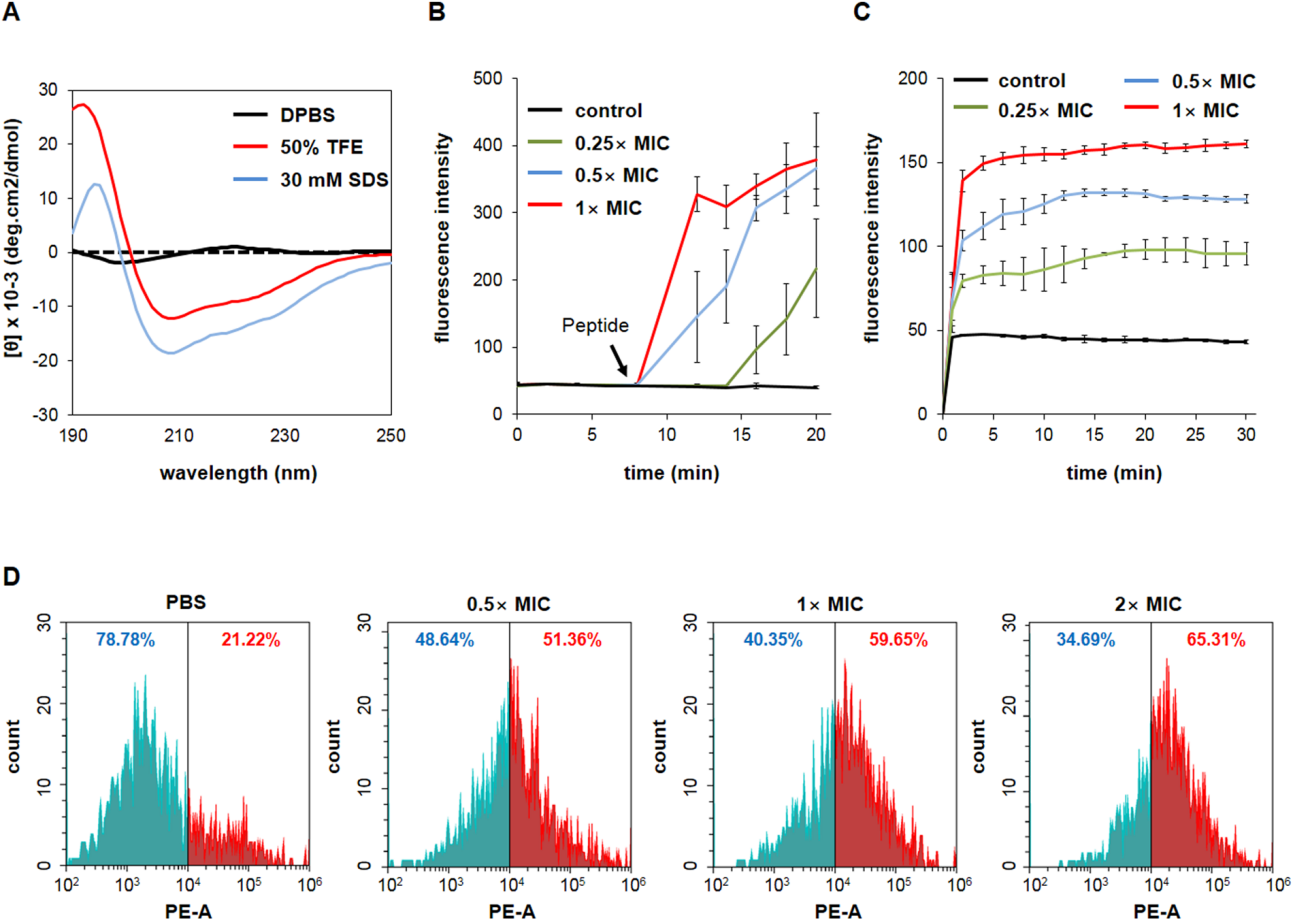
Disruption of the membrane integrity of *V. vulnificus* by HPA3P^His^ (**A**) The secondary structure of HPA3P^His^ was determined by CD spectroscopy at 40 μΜ in DPBS, 30 mM SDS, or 50% TFE. (**B**) The effect of HPA3P^His^ on the membrane depolarization of *V. vulnificus* MO6-24/0 was evaluated using the membrane-fluorescent sensitive dye DISC_3_-5. The bacteria were washed, resuspended to an OD_600_ of 0.05, and then mixed with DiSC_3_-5 (10 μΜ). HPA3P^His^ was added to the bacterial suspension and fluorescence was measured at an excitation wavelength of 622 nm and emission wavelength of 670 nm. (**C**) *V. vulnificus* MO6-24/0 (2 × 10^7^ CFU/ml) were incubated with 1 μΜ SYTOX Green for 15 min. The peptide was added to the bacterial suspension and the fluorescence intensity was monitored for 30 min at an excitation wavelength of 485 nm and emission wavelength of 520 nm. (**D**) Changes in bacterial membrane integrity during treatment with peptide were measured by flow cytometry after PI staining. *V. vulnificus* MO6-24/0 were washed and then adjusted to an OD_600_ of 0.2. The bacteria were treated with peptide for 5 min and then harvested by centrifugation. The bacterial pellets were mixed with PI (10 μg/ml) and then incubated for 15 min, followed by flow cytometric analysis.

The structural features of HPA3P^His^ in an aqueous solution of Dulbecco’s phosphate-buffered solution (DPBS), 30 mM sodium dodecyl sulfate (SDS), or 50% 2,2,2-trifluoroethanol (TFE) were studied using circular dichroism (CD). SDS can form micelles with a negatively charged surface and, thereby, mimic bacterial membranes, and TFE can induce a helical structure and stabilize secondary structures^23^. HPA3P^His^ exhibits as random coils in aqueous DPBS solution (Fig. 1A). By contrast, HPA3P^His^ showed an α-helical structure in solutions containing either SDS or TFE (Fig. 1A). These CD results suggest that HPA3P^His^ is capable to bind to bacterial membranes due to its electrostatic and hydrophobic interactions, imposing structural change of the membranes. Next, the ability of HPA3P^His^ to permeabilize intact membranes was analyzed using the membrane potential-sensitive dye 3,3′-dipropylthiadicarbocyanine iodide (DiSC_3_-5). Should antimicrobial peptides or any other compounds affect the potential of the bacterial membrane, the dye releases into the buffer, resulting increase in fluorescence^24^. Once the fluorescence intensity stabilized, *V. vulnificus* MO6-24/0 were treated with HPA3P^His^ at 0.25X, 0.5X, and 1X of the MIC. Treatment with HPA3P^His^ resulted in an immediate increase in fluorescence in a concentration-dependent manner, suggesting that HPA3P^His^ has membrane-depolarizing activity (Fig. 1B). We also examined whether the integrity of the bacterial membrane was disrupted by HPA3P^His^, using SYTOX Green and propidium iodide (PI). These dyes cannot pass through intact membranes, but will bind to DNA upon membrane damage by antimicrobial agents, resulting in an increase in fluorescence. The fluorescence intensity of SYTOX Green increased after treatment with HPA3P^His^, indicating the bacterial membrane was disrupted (Fig. 1C). We further confirmed the disruption of *V. vulnificus* membranes integrity by PI staining and flow cytometry. As shown in Fig. 1D, 21.22% of the bacteria in the *V. vulnificus* MO6-24/0 control group stained positive for PI. The groups treated with HPA3P^His^ showed the increased PI fluorescent signal in a concentration-dependent manner compared to the control (Fig. 1D). Collectively, these data indicate that HPA3P^His^ induces bacterial cell death through a membranolytic mechanism.

### Effective delivery of HPA3P^His^ in mammalian cells by AuNP-Apt^His^ conjugates

**Figure 2:**
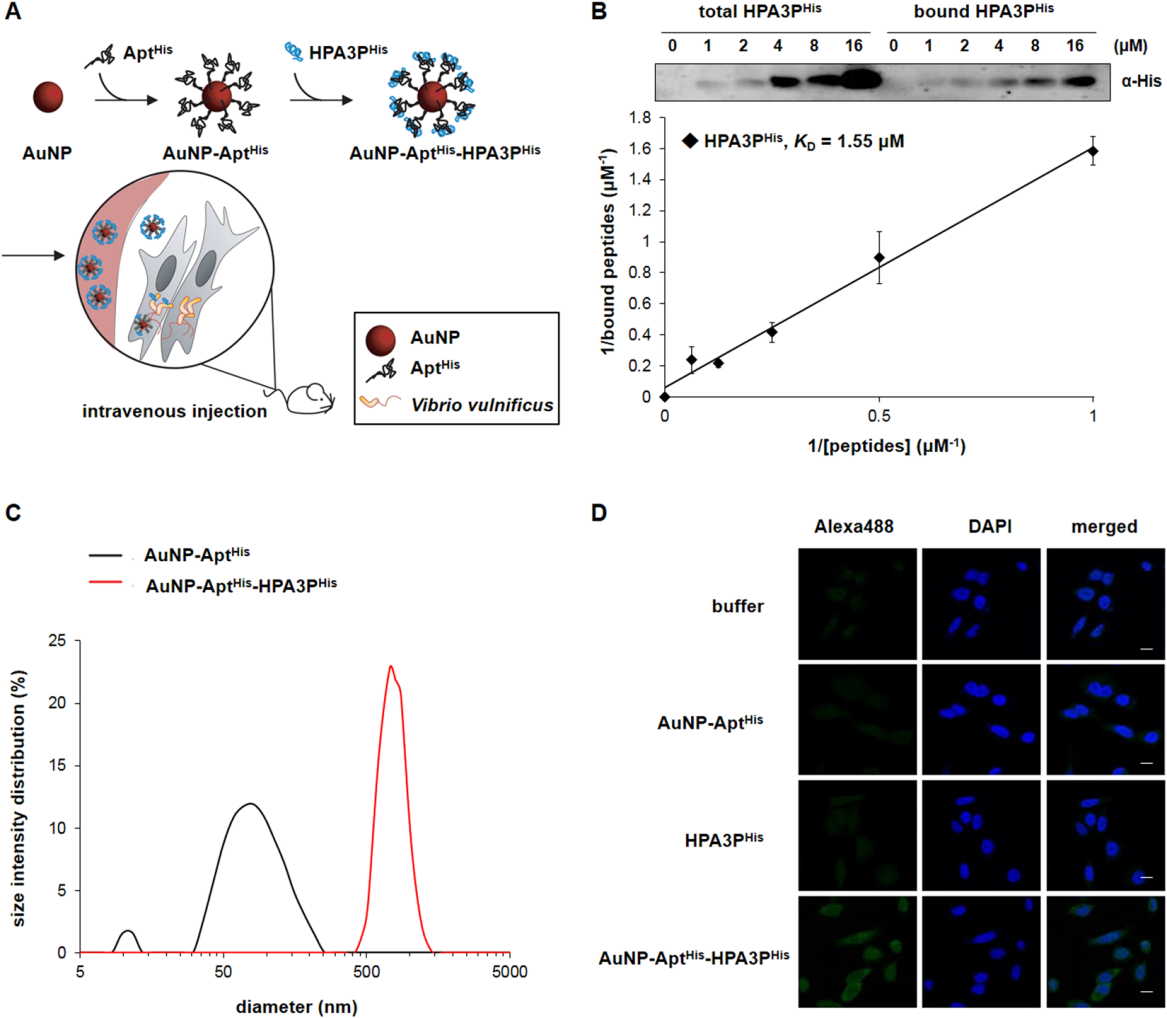
Preparation of AMP delivery system using AuNP-Apt^His^ conjugates (**A**) Schematic diagram illustrating the process of His-tagged HPA3P (HPA3P^His^) loaded on His-aptamer-functionalized AuNP (AuNP-Apt^His^) and the delivery of HPA3P^His^ to *Vibrio vulnificus*-infected host. (**B**) The binding capacity of AuNP-Apt^His^ to HPA3P^His^ was determined. The indicated concentrations of HPA3P^His^ were incubated with AuNP-Apt^His^ (1 nM), and AuNP-Apt^His^-bound HPA3P^His^ (bound HPA3P^His^) were analyzed by Tricine-SDS-PAGE and immunoblotting using His antibody. The amount of HPA3P^His^ bound to AuNP-Apt^His^ was analyzed by quantification of band intensities and compared with the standards where known amounts of HPA3P^His^ (total HPA3P^His^) were electrophoresed. The dissociation constant (*K*_D_) was obtained from the slope of the graphs presented. (**C**) The size distribution of AuNP-Apt^His^ and AuNP-Apt^His^-HPA3P^His^ analyzed by dynamic light scattering (DLS) is shown. (**D**) Representative confocal immunofluorescence images of HeLa cells treated with buffer, AuNP-Apt^His^, HPA3P^His^, or AuNP-Apt^His^-HPA3P^His^ for 10 min are presented. The cells were stained with rabbit anti-His followed by rabbit IgG-labeled with Alexa 488 (green), and the nuclei were stained with DAPI (blue). Scale bar = 20 μm.

HPA3P^His^ were loaded on AuNP-Apt^His^ by simple mixing, followed by incubation for 10 min to generate AuNP-Apt^His^-HPA3P^His^ composite (Fig. 2A). The binding capacity assay indicated efficient association of HPA3P^His^ with AuNP-Apt^His^, where approximately 50–60% of the HPA3P^His^ in the reaction mixture bound to AuNP-Apt^His^ (Fig. 2B). The particle size distribution data from the dynamic light scattering (DLS) assay demonstrated that the average diameters of AuNP-Apt^His^ and AuNP-Apt^His^-HPA3P^His^ were 83.0 ± 1.3 nm and 874.7 ± 232.8 nm, respectively (Fig. 2C). The zeta potential (ζ) of AuNP-Apt^His^ and AuNP-Apt^His^-HPA3P^His^ was −39.32 mV and −25.23 mV, respectively, indicating that the formation of AuNP-Apt^His^-HPA3P^His^ complex decreases the negative charge on the AuNP-Apt^His^ surface, and this decrease facilitates the cellular uptake of particles^25,26^. To examine the effectiveness of intracellular delivery of HPA3P^His^ by AuNP-Apt^His^, HeLa cells were incubated with AuNP-Apt^His^-HPA3P^His^ for 10 min, following which immunostaining of histidines and confocal laser scanning microscopic analyses were performed. As shown in Fig. 2D, strong fluorescent signals were detected in AuNP-Apt^His^-HPA3P^His^-incubated cells, whereas much weaker fluorescence was observed in cells incubated with HPA3P^His^ alone. These results indicate that AuNP-Apt^His^ composites efficiently deliver HPA3P^His^ into mammalian cells and that HPA3P^His^ alone does not effectively penetrate cells.

### Efficient bactericidal action of AuNP-Apt^His^-HPA3P^His^ leading to survival of *V. vulnificus*-infected host cells

**Figure 3:**
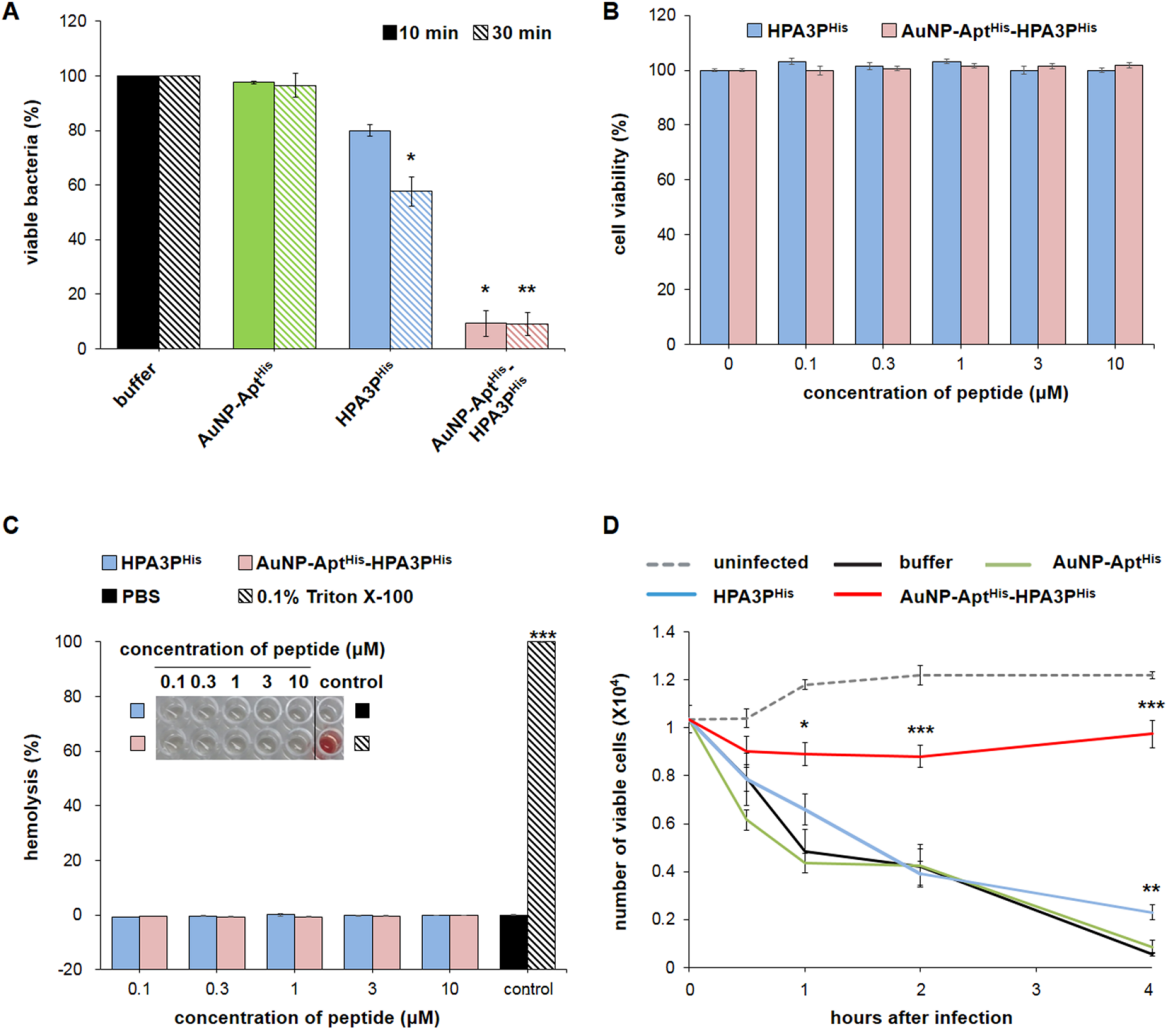
Protective effects of AuNP-Apt^His^-HPA3P^His^ on *V. vulnificus*-infected host cells by its effective bactericidal activity (**A**) *Vibrio vulnificus*-infected HeLa cells were incubated with buffer, AuNP-Apt^His^, HPA3P^His^, or AuNP-Apt^His^-HPA3P^His^ for 10 min or 30 min. Then, the viable intracellular *V. vulnificus* cells were counted and presented as % of viable bacteria normalized by the number of viable intracellular bacteria treated with buffer. The final concentrations of HPA3P^His^ and AuNP-Apt^His^ used were 0.5 μM and 1 nM, respectively. (**B**) The cytotoxicity of HPA3P^His^ or AuNP-Apt^His^-HPA3P^His^ on host cells was assessed by cell viability. HeLa cells were incubated with increasing concentrations of HPA3P^His^ or AuNP-Apt^His^-HPA3P^His^ for 24 h. (**C**) The hemolysis activities of HPA3P^His^ or AuNP-Apt^His^-HPA3P^His^ on red blood cells isolated from mice were measured as described in the Methods. PBS or 0.1% Triton X-100 were included as a negative or a positive control. (**D**) The protective effects of HPA3P^His^ or AuNP-Apt^His^-HPA3P^His^ on *V. vulnificus*-infected HeLa cells were assessed by measuring cell viability after incubation for indicated durations. All quantified data represents mean ± SEM from three independent experiments. Asterisks indicate statistically significant values (*p < 0.05; **p < 0.01; ***p < 0.001).

To examine the antibacterial effect of AuNP-Apt^His^-HPA3P^His^, *V. vulnificus*-infected HeLa cells were incubated with AuNP-Apt^His^-HPA3P^His^ for 10 or 30 min. The viable intracellular *V. vulnificus* cells were extracted from HeLa cells and analyzed by colony-forming assay. Compared with that of buffer- or AuNP-Apt^His^-incubated cells, the number of viable *V. vulnificus* cells in the AuNP-Apt^His^-HPA3P^His^-treated cells decreased by 90% in 10 min (Fig. 3A). In contrast, only 20% or 40% decrease in viable *V. vulnificus* cells after for 10 or 30 min of treatment with HPA3P^His^ was observed, respectively (Fig. 3A). HPA3P^His^ and AuNP-Apt^His^-HPA3P^His^ did not exhibit cytotoxicity against HeLa cells and mouse erythrocytes up to 10 µM as determined by cell viability and hemolysis assays, respectively (Fig. 3B and C). In addition, we determined the effect of AuNP-Apt^His^-HPA3P^His^ on *V. vulnificus*-infected host cell survival. *V. vulnificus*-infected HeLa cells were incubated with buffer, AuNP-Apt^His^, HPA3P^His^, or AuNP-Apt^His^- HPA3P^His^, and viable HeLa cells were measured up to 4 h. Nearly all the infected HeLa cells incubated with buffer, AuNP-Apt^His^, or HPA3P^His^ were dead at 4 h after infection (Fig. 3D). In contrast, the treatment of infected HeLa cells with AuNP-Apt^His^-HPA3P^His^ completely prevented their death (Fig. 3D). Thus, these results suggest that AuNP-Apt^His^ conjugates improve the penetrability of HPA3P^His^, leading to the effective bactericidal action of HPA3P^His^.

### Prevention of death of *V. vulnificus*-infected mice by AuNP-Apt^His^-HPA3P^His^ treatment

**Figure 4:**
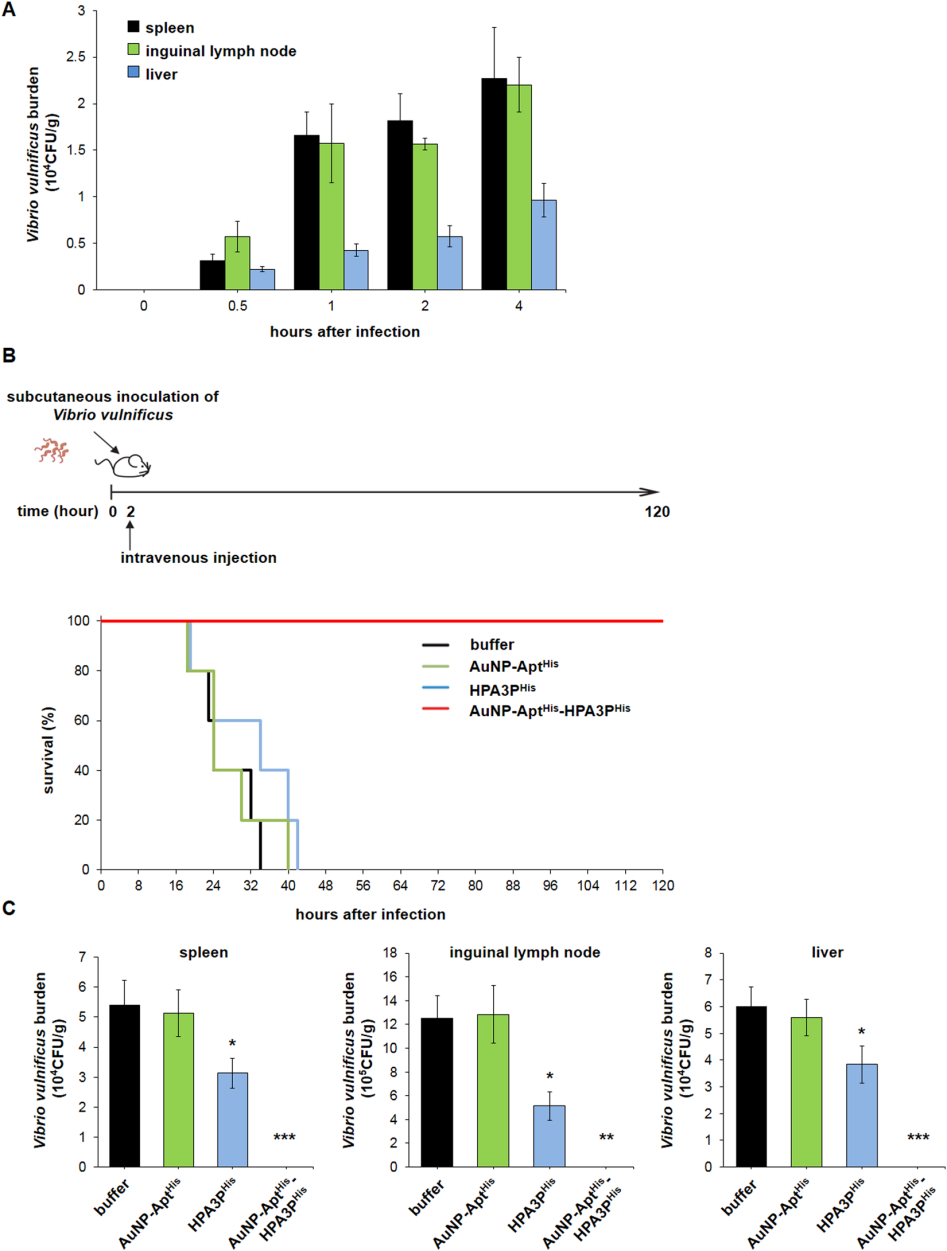
Complete protection from mortality induced by *Vibrio vulnificus* infection in mice by systemic administration of AuNP-Apt^His^-HPA3P^His^ (**A**) Distribution of *V. vulnificus* in the organs of mice after subcutaneous inoculation of *V. vulnificus* (5 × 10^4^ CFU). The mice were sacrificed at indicated time points. Each data point represents the mean ± SEM of two mice. (**B**) Schematic illustration of *in vivo* mice experiment is presented at the top. The percent survival of *V. vulnificus*-infection mice after treatment with buffer, AuNP-Apt^His^ (5 nM), HPA3P^His^ (2 mg/kg), or AuNP-Apt^His^-HPA3P^His^ (~1 mg/kg of AMP loaded on 5 nM of AuNP-Apt^His^) until 120 h after infection has been presented. Five mice were used for each group. (**C**) The *in vivo* bactericidal efficacy of AuNP-Apt^His^-HPA3P^His^ was confirmed by examining the viable *V. vulnificus* counts in the spleen, inguinal lymph node (ILN), and liver isolated from the *V. vulnificus*-infected mice treated with buffer, AuNP-Apt^His^, HPA3P^His^, or AuNP-Apt^His^- HPA3P^His^. Four mice were used for each group, and mice were sacrificed 18 h after *V. vulnificus* infection. Data are expressed as the number of CFUs/g organ. Asterisks indicate statistically significant values (*p < 0.05; **p < 0.01; ***p < 0.001).

We extended our study to test the effectiveness of AuNP-Apt^His^-HPA3P^His^ *in vivo* in mice. To induce septicemia and wound infection caused by the invasion of *V. vulnificus* into tissues and vasculature, *V. vulnificus* was subcutaneously inoculated into the skin of mice^27^. The spreading of *V. vulnificus* in the organs of mice, including spleen, inguinal lymph node, and liver, was monitored for 4 h after inoculation. As shown in Fig. 4A, *V. vulnificus* sufficiently colonized in these organs as early as 30 min. Using this mouse model, the *in vivo* therapeutic effect of AuNP-Apt^His^-HPA3P^His^ was examined. Mice were infected with *V. vulnificus*, followed by a single intravenous injection with buffer, AuNP-Apt^His^, HPA3P^His^, or AuNP-Apt^His^-HPA3P^His^ at 2 h after inoculation. Then, the survival rate of the mice was observed for 120 h. All infected mice injected with buffer, AuNP-Apt^His^, or HPA3P^His^ died before 42 h after infection (Fig. 4B). In sharp contrast, all the infected mice injected with AuNP-Apt^His^-HPA3P^His^ survived until 120 h (Fig. 4B). To ensure that the survival of mice was due to the inhibition of *V. vulnificus* by HPA3P^His^ delivered by AuNP-Apt^His^, the number of viable *V. vulnificus* cells was measured in the spleen, inguinal lymph node, and liver homogenates of these mice at 18 h after inoculation. There was no colonization of *V. vulnificus* in the AuNP-Apt^His^-HPA3P^His^-treated mice organs, whereas the organs from the mice injected with buffer, AuNP-Apt^His^, or HPA3P^His^ showed significant colonization of *V. vulnificus* (Fig. 4C).

## Discussion

In this study, we demonstrated that AuNP-Apt^His^-HPA3P^His^ is a potent antimicrobial agent against *V. vulnificus* infection, supported by its rapid and highly effective bactericidal action in host cells. *V. vulnificus* is a representative pathogenic strain that evades the host defense systems and replicates rapidly, resulting in mortality among humans and aquatic animals. Its infection contributes to 95% of seafood-related mortality in humans^9,28^. A high dose of combinational antibiotics, such as doxycycline + ceftazidime, is a common primary therapeutic regimen against *V. vulnificus* infection^29^. However, studies on antibiotic sensitivity of *V. vulnificus* isolates from the US, Europe, and Asia indicate that substantial portions of the isolates show resistance to various antibiotics^9^. This emergence of multidrug-resistant *V. vulnificus* imposes the need to seek alternative therapeutic approaches.

Naturally occurring AMPs are produced as gene-encoded precursor proteins by all living organisms from prokaryotes to humans to protect the host from infections and inhibit competing microbes in bacteria^11^. The interaction of amphipathic AMPs with bacterial membrane disrupts the integrity of the membrane, leading to rapid membrane permeabilization and lysis of bacteria^30,31^. Structural and functional diversities of AMPs along with their rapid mode of action render AMPs attractive pharmacological agents that could overcome antibiotic resistance. Few AMPs are already in the market and various AMPs are currently under clinical trial as antimicrobial agents^11,12^. However, the currently used AMPs are largely limited to topical applications due to their rapid proteolytic degradation and clearance as peptides in addition to their ineffective *in vivo* systemic delivery^11,32^.

Although researchers have mainly focused on identifying bactericidal AMPs and their derivatives in the last decade, limited efforts have been made for innovative development to enhance the effectiveness of AMPs in living systems. AMPs, being cationic or amphiphilic, can readily bind to serum proteins and are degraded by proteolytic enzymes in the blood plasma, followed by rapid clearance from circulation, resulting in loss of their antimicrobial activity^32–34^. To prevent AMP proteolysis, elaborative chemical modifications of AMPs, such as generation of prodrug, cyclization of AMPs, and tagging or blocking of amino- or carboxyl ends of AMPs, have been attempted^35–38^. More recently, various AMP formulations with nanocarriers, such as lipids and polymers, were evaluated to increase the efficacy of AMPs; however, usually the process of loading AMPs is complex and their *in vivo* effectiveness was minimal or not confirmed^12,32^. Currently, only a few reports on the development of bactericidal AMPs against *V. vulnificus* infection are available^39–43^.

To the best of our knowledge, we are the first to develop an AMP-derived antibacterial agent with high efficacy in *V. vulnificus*-infected mammal. A single intravenous (IV) injection of AuNP-Apt^His^-HPA3P^His^ in *V. vulnificus*-infected mice efficiently and rapidly eliminates *V. vulnificus* in the host, as evidenced by no viable *V. vulnificus* in the organs of mice at 18 h after injection (Fig. 4C). All the infected mice survived after the administration of AuNP-Apt^His^-HPA3P^His^, which was in sharp contrast to 0% survival rate among control mice (Fig. 4B). The intravenous injection of HPA3P^His^ alone failed to prevent death of *V. vulnificus*-infected mice, resulting in 100% mortality (Fig. 4B). These results indicate that this robust bactericidal activity observed is mainly attributable to the conjugation of HPA3P^His^ to AuNP-Apt^His^. The functional contribution of AuNP-Apt^His^ is that it enables the efficient intracellular delivery of HPA3P^His^ to the host in addition to the increase in stability of HPA3P^His^ by protecting HPA3P^His^ from proteolysis^21^. Another advantageous property of this system lies in its simplicity to be employed in a wide range of applications, where AuNP-Apt^His^ conjugates can load any type of AMPs after His-tagging by simple mixing for few minutes at room temperature. Moreover, AuNP-Apt^His^ conjugates were effective after a single administration, and thus, this longer lasting efficacy is a useful parameter that can help in avoiding short-term repetitive administration and reducing overall cost for therapy. Additionally, AuNP-Apt^His^-HPA3P^His^ did not exhibit any evident host toxicity (Figs 3B,C and 4B). Their efficient intracellular uptake and effective bactericidal activity would contribute to reduced dosing regimen, which renders minimizing possible toxic response. Earlier studies reported that AuNPs exhibit little toxicity, which is largely dependent on the particle size^44,45^. 15-nm AuNPs used in this study have known to be non-cytotoxic^20,46,47^. Thus, these advantageous properties of AuNP-Apt^His^-derived AMP delivery system render it a promising antibacterial therapeutic candidate. To facilitate the development of AuNP-aptamer-AMP conjugate-based therapeutics, pre-clinical studies that prove the safety of AuNP-aptamer composites as well as the assessment of economic feasibility for large-scale production of AMPs are necessary. In addition, massive bactericidal screening of other candidate AMPs using HPA3P^His^ as a reference would contribute to development of more effective AuNP- aptamer-AMPs as antimicrobial therapeutics.

## Methods

### Peptide synthesis and purification

The peptides were synthesized using the 9-fluorenylmethoxycarbonyl (Fmoc) solid-phase method on Rink amide 4-methyl benzhydrylamine resin (Novabiochem; 0.55 mmol/g) with a Liberty microwave peptide synthesizer (CEM Co., Matthews, NC, USA). The peptides were prepared according to a previously described method^21^.

### Antibacterial assay

*V. vulnificus* MO6-24/O, *S. typhimurium* and *S. aureus* cells were cultured at 37 °C in cation adjusted Mueller-Hinton broth (CA-MHB; BD Difco™, Detroit, MI, USA). MIC and MBC of each AMP was measured in microdilution assays according to Clinical and Laboratory Standards Institute (CLSI) recommendations^48^. In brief, serial two-fold dilutions of 0.5–64 μM of each AMP were added to the CA-MHB media containing cultures of mid-log phase bacteria (5 × 10^5^ CFUs/ml). The cultures were grown for an additional 16–24 h at 37 °C. After incubation, MIC and MBC were determined as the lowest concentrations of AMP inhibiting bacterial growth based on the OD_600_ measurements or required to kill 99.9% of the test inoculum, respectively.

### Circular dichroism (CD) spectroscopy

CD spectroscopy was performed using a Jasco-810 spectrapolarimeter (Jasco, Tokyo, Japan), with a quartz cell with a 1.0-mm path length. Spectra were measured at wavelengths ranging from 190 to 250 nm. CD spectral data of the peptide at a fixed concentration of 40 µΜ were recorded in Dulbecco’s phosphate-buffered solution (DPBS), 30 mM sodium dodecyl sulfate (SDS) solution, or 50% 2,2,2-trifluoroethanol (TFE) solution.

### Membrane depolarization assay

The effect of HPA3P^His^ on bacterial membrane depolarization was measured using the membrane potential-sensitive dye DiSC_3_-5. *V. vulnificus* MO6-24/0 was cultured in LBS medium at 30 °C and then washed three times with buffer A (DPBS containing 20 mM glucose). The bacteria were resuspended to an OD_600_ of 0.05 in buffer A with 0.1 M KCl added and then DiSC_3_-5 was added to a final concentration of 0.1 µΜ. This mixture was incubated for 15 min to stabilize the level of fluorescence. Different concentrations, 0.25X, 0.5X, and 1X of the MIC of HPA3P^His^ and DPBS were added. The fluorescence was continuously measured for 10 min at an excitation wavelength of 622 nm and emission wavelength of 670 nm.

### SYTOX Green uptake assay

*V. vulnificus* MO6-24/0 were grown in LBS medium at 30 °C, washed, and then resuspended in DPBS to 2 × 10^7^ CFU/ml. After adding 1 µΜ SYTOX Green, the bacteria were incubated for 15 min in the dark. HPA3P^His^ was added at 0.25X, 0.5X, and 1X of the MIC to the bacterial suspensions. Changes in fluorescence as a result of the interactions between SYTOX Green and bacterial DNA were measured at an excitation wavelength of 485 nm and emission wavelength of 520 nm.

### Flow cytometric analysis of bacterial membrane disruption

The disruption of *V. vulnificus* membranes by the peptide was analyzed by flow cytometry. *V. vulnificus* MO6-24/0 were cultured in LBS medium and harvested by centrifugation at 6000 g for 10 min. After washing with DPBS, the bacteria were resuspended to an OD_600_ of 0.2, and then treated with 0.5X, 1X, or 2X of the MIC of HPA3P^His^ and DPBS for 5 min at 30 °C with shaking at 140 rpm. After the treatment, the bacteria were harvested and incubated with 10 μg/ml of PI for 20 min. Bacterial cell staining with PI was measured using a CytoFLEX flow cytometer (Beckman, Brea, CA, USA).

### Preparation of AuNP-Apt conjugates and AuNP-Apt-AMP complex

Citrate-stabilized AuNPs (15 nm diameter) were purchased from BBI Life Science (UK). His-tag DNA aptamers^20^ were conjugated to AuNPs by a previously described method^49^ and the AuNP-Apt-AMP complex was prepared according to a previously described procedure^21^. The size and ζ-potential of AuNP-Apt-AMP were investigated using a dynamic light scattering spectrophotometer (ELSZ-1000; Otsuka Electronic Korea, Seongnam, Korea).

### Binding capacity assay

Binding capacity assay between AMPs and AuNP-Apt conjugates was performed according to a previously described method^20^.

### Mammalian cell culture

HeLa cells were cultured in DMEM (Welgene, Daegu, Korea) supplemented with 10% fetal bovine serum (Welgene) and 1% penicillin–streptomycin (Welgene) at 37 °C/5% CO_2_.

### Visualization of peptide delivered by AuNP-Apt conjugates to mammalian cells

HeLa cells (5 × 10^4^/well) were seeded on 10-mm cover slips and incubated with the AuNP-Apt-AMP complex and control reagents for 10 min. The final concentration of AMP and AuNP-Apt conjugate was 0.5 μM and 1 nM, respectively. The cells were fixed and immunostained as previously described^21^.

### Cytotoxicity assay

The cytotoxicity of AMP against HeLa cells was determined as previously described^21^.

### Viable intracellular bacterial count assay

HeLa cells (5 × 10^4^/well) were cultured for 18–24 h in 24-well culture dishes. HeLa cells were infected with *V. vulnificus* MO6-24/O cells for 30 min at a multiplicity of infection (MOI) of 20. The infected cells were washed with DMEM containing 50 mg/ml gentamicin (Gibco, Invitrogen, Carlsbad, CA, USA) and PBS. Then, cells were treated with AuNP-Apt-AMP complex or control reagents for 30 min. The HeLa cells were washed with PBS and lyzed with PBS containing 1% (v/v) Triton X-100, and intracellular bacteria were plated onto LBS plates to determine the numbers of CFUs.

### V. vulnificus-infected mammalian cell viability assay

HeLa cells (1 × 10^4^/well) were seeded in 96-well culture dishes. After 18–24 h, the HeLa cells were treated with buffer or infected with *V. vulnificus* for 30 min at a 1:20 MOI. The cells were washed with 50 mg/ml gentamicin (Gibco, Invitrogen). Next, the cells were incubated for 0, 0.5, 1, 2, and 4 h with AuNP-Apt-AMP complex or control reagents. The infected cells were harvested by trypsin-ethylenediaminetetraacetic acid, and viable HeLa cells were measured by trypan blue exclusion assay.

### Animals

Animal studies were performed on specific-pathogen-free, 6-week-old, female ICR mice (DBL, Eumsung, Korea) weighing 18–20 g. Mice were housed in a room under a 12 h reversed light cycle, humidity of 30–40%, and temperature of 22 ± 1 °C. The experiment protocols were approved by the Chung-Ang University Support Center for Animal Experiments, and all methods were performed in accordance with the guidelines and regulations.

### Hemolysis assay

Fresh red blood cells (RBCs) from ICR mice (DBL) were collected and washed three times with PBS. The various concentrations (0.1 to 10 μM) of peptides were incubated with 8% (v/v) washed RBCs for 1 h at 37 °C. The samples were centrifuged at 800 × g for 10 min, and the absorbance of the supernatants was measured at 540 nm by microplate reader (ASYS UVM 340; Biochrom, Cambridge, UK). As a negative or a positive controls, PBS or 0.1% (v/v) Triton X-100 were used, respectively. The percentage of hemolysis was calculated by the following equation: % hemolysis = [(A_peptide_−A_PBS_)/(A_0.1% Triton X-100_−A_PBS_)] × 100

### *In vivo* mice experiment

*V. vulnificus MO6-24/O* cells grown overnight in LBS medium at 30 °C were inoculated and subsequently harvested when bacterial growth reached mid-log phase and washed with PBS. Mice were deprived of food and water for 4–18 h before inoculation. Then, mice were subcutaneously injected with 100 μl of PBS containing 1–5 × 10^4^ CFU of *V. vulnificus*. Two hours after bacterial challenge, *V. vulnificus*-infected mice were intravenously injected with AuNP-Apt^His^-HPA3P^His^ complex and control reagents. Mice were monitored for 120 h after injection and immediately killed when they were moribund.

### Viable bacterial count in murine organs

At 18 h after bacterial challenge, mice of each group were sacrificed and the spleen, inguinal lymph node, and liver were removed. The organs were weighed, homogenized, and dissolved in LBS. The homogenates were plated on LBS agar and the plates were incubated at 30 °C overnight. Results were expressed as the number of CFUs/g.

### Statistical analysis

Data were presented as means ± SEM. Student’s *t*-test (Excel; Microsoft Corp., Redmond, WA, USA) was used, and *p* < 0.05 was considered statistically significant.

### Availability of materials and data

The authors declare no restrictions on the availability of materials or information.
